# Adjunctive hyperbaric oxygen therapy for chronic diabetic foot ulcer unresponsive to standard care: A case report

**DOI:** 10.37796/2211-8039.1693

**Published:** 2026-03-01

**Authors:** Sukriyadi Adi, Ismail Ismail, Sitti Rahmatiah, Agustan Agustan

**Affiliations:** a Nursing Department, Makassar Health Polytechnic, South Sulawesi, Indonesia; b Dr. Wahidin Sudirohusodo Central General Hospital, Makassar, South Sulawesi, Indonesia

**Keywords:** Diabetic foot ulcer, Hyperbaric oxygen therapy, Wound healing

## Abstract

**Background:**

Diabetic foot ulcers (DFUs) are a serious complication of diabetes mellitus and are often challenging to treat, particularly in patients who fail to respond to standard wound care. Adjunctive therapies such as hyperbaric oxygen therapy (HBOT) have shown potential in promoting wound healing in chronic cases.

**Case presentation:**

A 66-year-old male with a 10-year history of type 2 diabetes mellitus presented with a chronic, non-healing DFU on the right foot. Despite oral antibiotic therapy and conventional wound management, the ulcer demonstrated progressive necrosis, persistent infection, and tissue exposure. Wound culture identified Klebsiella oxytoca. After targeted antibiotic treatment, the patient underwent 35 sessions of HBOT at 2.4 ATA for 90 min per session, five days per week, in conjunction with hydrogel dressings and structured wound care strategies.

**Results:**

Over the treatment course, the wound exhibited substantial clinical improvement, including reduced edema, infection control, emergence of granulation tissue, and near-complete epithelialization. No complications occurred during HBOT, and the patient tolerated the therapy well.

**Conclusion:**

This case demonstrates the potential benefits of HBOT as an adjunctive therapy in chronic DFUs unresponsive to standard care. HBOT, when combined with modern wound care strategies, may accelerate healing and reduce the risk of amputation in select patient populations. Further research is warranted to refine treatment protocols and establish evidence-based criteria for patient selection.

## 1. Introduction

Diabetic foot ulcers (DFUs) are among the most serious and debilitating complications of diabetes mellitus, affecting approximately 15 % of individuals with diabetes during their lifetime and accounting for the majority of diabetes-related lower-limb amputations worldwide [1,2]. Chronic DFUs not only reduce the quality of life for patients but also impose a substantial socioeconomic burden due to prolonged hospitalizations, increased risk of infection, and complex treatment needs [3].

Standard management strategies for DFUs include glycemic control, regular debridement, infection management, and moisture-balanced wound dressings [4]. However, in many cases complicated by ischemia, polymicrobial infection, or persistent tissue hypoxia, healing may be delayed or fail entirely despite appropriate conventional care [5]. This highlights the need for adjunctive therapies that can enhance oxygenation, modulate inflammation, and promote tissue regeneration.

Hyperbaric oxygen therapy (HBOT) has emerged as a promising adjunctive modality for nonhealing DFUs. HBOT involves delivering 100 % oxygen in a pressurized chamber (typically 2.0–2.5 ATA), which significantly increases plasma oxygen concentration and enhances delivery to ischemic tissues. This process supports neovascularization, fibroblast proliferation, collagen synthesis, and leukocyte-mediated bacterial clearance mechanisms that are vital for effective wound healing [6].

Several clinical trials and meta-analyses support the use of HBOT for diabetic foot ulcers, demonstrating improved wound healing, reduced amputation rates, and enhanced infection control when used in conjunction with standard therapy [7–9]. However, the literature also highlights variability in treatment protocols, patient selection criteria, and outcome measures, underscoring the importance of individualized care and real-world case evaluations [10].

Recent advancements in wound care materials have significantly improved the therapeutic landscape for chronic wounds. For instance, Zhang et al. [11] developed multifunctional hydrogel dressings integrating antimicrobial and angiogenic properties to promote diabetic wound healing. Likewise, Wang et al. [12] introduced a bioactive composite material that accelerates tissue regeneration through immunomodulation and sustained oxygen delivery. Meanwhile, Li et al. [13] demonstrated the synergistic effect of exosome-loaded scaffolds on cellular migration and granulation tissue formation in diabetic wounds.

While these materials-based approaches offer promising innovations in experimental settings, their translation into routine clinical practice particularly in resource-limited clinical settings. The present case study builds upon these findings by showcasing a real-world application of adjunctive HBOT in combination with hydrogel therapy for a chronic DFU unresponsive to standard care.

Unlike prior material-based studies, this report emphasizes practical integration, treatment monitoring, and patient-specific adaptation of HBOT protocols, providing valuable clinical insight complementary to ongoing technological advances in wound healing research.

## 2. Case report

A 66-year-old Indonesian male with a 10-year history of type 2 diabetes mellitus, managed with oral metformin (850 mg/day), presented in August 2024 with a non-healing DFU located on the dorsum and lateral aspect of the right foot. The ulcer had progressively worsened over four weeks prior to presentation. The patient had no known family history of diabetes or peripheral vascular disease.

Initial evaluation at Dr. Wahidin Sudirohusodo Central General Hospital, Makassar, revealed an ulcer with irregular margins, exposed subcutaneous tissue, necrotic slough, and purulent discharge. The surrounding skin was edematous, erythematous, and discolored suggestive of cellulitis with potential progression toward necrotizing soft tissue infection. A foul-smelling, serosanguinous exudate was noted, and the patient reported severe localized pain.

**Fig. 1:**
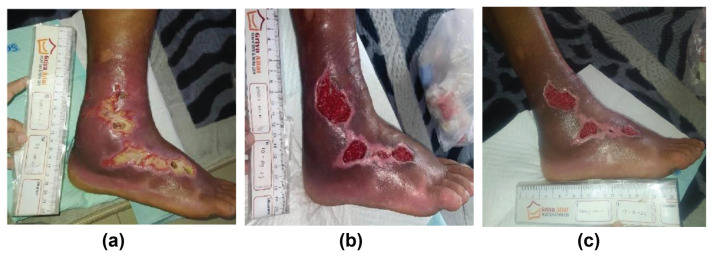
Serial wound images documenting healing progression with HBOT: **(a)** Initial presentation with necrotic tissue, edema, and purulent discharge, **(b)** Mid-therapy image showing granulation tissue and reduced infection, **(c)** Final therapy image indicating over 90 % epithelial coverage and minimal exudate. Images were obtained under standardized lighting, with informed patient consent.

Initial treatment consisted of oral ciprofloxacin (500 mg twice daily), paracetamol (500 mg as needed), and local wound care using topical antibiotic ointment (Wound Zalf^1^) with gauze dressings and antiseptic irrigations. However, there was no improvement after several weeks of adherence to this regimen (Fig. 1a).

In September 2024, the patient was referred to the hospital’s Hyperbaric Oxygen Therapy (HBOT) Unit. Laboratory analysis showed persistent hyperglycemia, baseline glycemic control was suboptimal, with an HbA1c of 9.2 %. Wound culture identified Klebsiella oxytoca. The antibiotic regimen was adjusted to sulfamethoxazole (400 mg) and trimethoprim (80 mg) twice daily for seven days. In response, a multidisciplinary team implemented strict glycemic control measures, including dietary modifications, daily glucose monitoring, and titration of metformin to 1000 mg/day. Nutritional counseling was provided by a clinical dietitian. HBOT was administered at 2.4 ATA for 90 min per session, five times per week, totaling 35 sessions over a seven-week period. While standard HBOT regimens typically include 20–30 sessions, the extended course was based on ongoing clinical improvements, particularly the emergence of granulation tissue and continued epithelialization observed after the 25th session. This individualized approach was aligned with patient-specific response criteria, which monitored through weekly assessments.

The patient was closely monitored for HBOT-related adverse events, including barotrauma, oxygen toxicity, and otic discomfort. Standard pre-treatment assessments and post-session evaluations were conducted. No complications or adverse effects were reported during or following therapy.

By the third week of HBOT, the wound showed marked improvement with decreased edema, reduced necrotic slough, and early granulation tissue formation. Epithelialization was visible at the wound edges, and partial contraction had begun. Edema and shiny skin over the right instep and ankle persisted, likely due to lymphatic congestion, though there were no signs of active infection (Fig. 1b).

At session 26, the wound bed was covered with well-vascularized granulation tissue and minimal fibrin. By the end of session 35 in October 2024, over 90 % of the wound surface was epithelialized, with mature epithelial tissue and no purulence or odor (Fig. 1c). Serial photographic documentation was obtained at baseline, mid-treatment, and post-treatment under standardized conditions, with informed consent. Clinical progression was documented through serial digital photography captured at baseline, during week three, and upon completion of therapy (Fig. 1a—c), with patient consent obtained for image use. These images provide direct visual evidence of wound evolution. Histological analysis was not performed, as it was not part of routine wound management in this clinical setting.

No complications were observed during HBOT, and the patient tolerated all sessions well without treatment interruption. The patient was routinely monitored for potential side effects such as oxygen toxicity, middle ear barotrauma, and confinement anxiety. He tolerated all sessions well, and there were no treatment interruptions (Tables 1 and 2).

## 3. Discussions

This case illustrates the successful use of HBOT as an adjunctive therapy in the management of a chronic DFU that had failed to respond to conventional wound care and oral antibiotics. The ulcer was characterized by progressive necrosis, persistent infection with Klebsiella oxytoca, and poor granulation tissue formation, which prompted escalation of therapy. Integration of HBOT resulted in clear clinical improvement, with resolution of infection, formation of robust granulation tissue, and progressive epithelialization of the wound. These outcomes support the role of HBOT in promoting tissue regeneration and infection control in recalcitrant diabetic wounds. While HBOT is generally safe, especially when administered in controlled clinical settings, prolonged treatment courses warrant careful surveillance. In this case, the decision to extend to 35 sessions was guided by progressive wound healing and absence of plateau in clinical response. The patient tolerated the therapy well, with no observed complications such as oxygen toxicity, barotrauma, or middle ear barotrauma, which are among the most commonly reported side effects of prolonged HBOT use. Routine otoscopic exams and symptom checklists were used to ensure treatment safety throughout the course.

Several studies have documented similar outcomes. Löndahl et al. [14] and Bajuri and Hassan [15] demonstrated improved healing in patients treated with HBOT compared to placebo, reporting a 52 % healing rate versus 29 % in the control group (p = 0.03). Salama et al. [8] and Hatibie et al. [16] also reported positive outcomes, with one-third of patients achieving complete healing under HBOT. Our case aligns with these findings, particularly as HBOT was applied in conjunction with wound debridement, targeted antibiotics, and hydrogel dressings.

Meta-analyses by Sharma et al. [7,17] further validate HBOT’s utility, showing reduced amputation rates and enhanced healing in DFU cases. The extended duration of therapy in this case (35 sessions) deviates from the more commonly reported range of 20–30 sessions but was justified based on sustained wound response and progressive epithelialization through the final weeks of treatment. The absence of complications monitored through otoscopic exams and side effect screening supports the safety of this extended protocol.

Nonetheless, conflicting evidence exists. Fedorko et al. [10] and Thistlethwaite KR et al. [18] reported no significant difference between HBOT and placebo in certain controlled trials. O’Reilly et al. [19] attributed such discrepancies to inconsistent patient selection, protocol variations, and methodological heterogeneity. These limitations reinforce the importance of individualized case assessment. In the present report, the chronic nature of the ulcer, documented bacterial infection, poor response to prior care, and visual progression captured through serial photography offered strong clinical indications for HBOT.

The use of hydrogel-based dressings as part of modern wound care strategy may have further contributed to wound bed hydration and epithelial migration [20–22]. While this combination remains underexplored in randomized trials, recent narrative and experimental reviews suggest that hydrogel—HBOT synergy holds potential for enhancing outcomes and minimizing complications such as maceration [23,24].

The differential diagnosis initially included cellulitis, severe soft tissue infection, and early necrotizing fasciitis, all of which carry high risks of limb loss if not aggressively managed. The detection of Klebsiella oxytoca guided specific anti-microbial therapy, which, in conjunction with HBOT, contributed to clinical resolution.

The prognosis in this case is favorable. After 35 HBOT sessions, the patient exhibited nearly complete epithelialization, reduced edema, and no signs of recurrent infection. Early referral for HBOT particularly in chronic non-healing DFUs may improve outcomes and reduce healthcare costs associated with extended hospitalizations and amputations.

Preventive strategies remain essential to reduce DFU incidence and complications. These include regular foot assessments, patient education, glycemic control, and early intervention upon wound detection. Adjunctive HBOT should be considered in patients with ulcers unresponsive to standard wound care after appropriate infectious and vascular assessments.

This case also underscores the critical role of glycemic control in wound healing. The patient’s elevated HbA1c at baseline (9.2 %) was addressed through dietary modification, increased metformin dosage, and frequent blood glucose monitoring. Improved glycemic control likely contributed to the wound’s healing trajectory, reinforcing the need for multidisciplinary diabetes management.

Ultimately, early identification of nonhealing ulcers and timely referral for HBOT, when combined with comprehensive wound care and metabolic optimization, may reduce hospitalization durations and lower the risk of major amputation. This case exemplifies the importance of real-world, patient-specific care strategies in chronic DFU management strategies.

## 4. Conclusion

This case highlights the clinical benefit of HBOT as an adjunctive intervention for a chronic DFU unresponsive to standard care. The integration of HBOT with structured wound management and hydrogel-based dressings resulted in substantial improvement, including infection resolution, robust granulation tissue formation, and near-complete epithelialization without complications. These outcomes underscore HBOT’s potential to enhance healing trajectories and reduce the risk of amputation in carefully selected patients. The findings reinforce the importance of individualized therapy, interdisciplinary coordination, and the timely escalation of care in chronic wound management.

## Figures and Tables

**Table 1:** Wagner classification staging of diabetic foot ulcer throughout HBOT.

Evaluation Time Point	Wagner Grade*	Clinical Description
August 2024 (Initial Visit)	Grade 2*	Deep ulcer reaching tendon or joint capsule, with extensive necrotic tissue and surrounding cellulitis.
September 10, 2024 (Before HBOT)	Grade 3*	Deep ulcer with abscess formation and infection confirmed by Klebsiella oxytoca culture.
September 3rd Week (During HBOT)	Grade 2*	Ulcer depth reduced; granulation tissue forming with controlled infection and less erythema.
October 2024 (Final HBOT Session)	Grade 1*	Superficial ulcer with healthy granulation and epithelialization; no signs of active infection.

* Wagner Classification used to objectively assess ulcer severity and progression throughout hyperbaric oxygen therapy (HBOT).

The case progressed from a deep infected ulcer with abscess (Grade 3) to a superficial epithelializing ulcer (Grade 1), confirming clinical improvement with adjunctive HBOT.

**Table 2:** Clinical wound assessment during HBOT.

Time Point	Ulcer Depth & Tissue	Infection Status	Granulation & Epithelialization	Wound Edges & Size	Surrounding Skin Condition
August 2024 (Initial Visit)	Deep ulcer, exposed subcutaneous tissue	Cellulitis, possible necrotizing fasciitis	Minimal granulation; necrosis dominant	Irregular, large ulceration	Edema, purplish discoloration, inflamed
September 10, 2024 (Before HBOT)	Increased depth, necrotic slough	Klebsiella oxytoca identified; pus present	Necrosis worsening	Expanding wound margins, excess drainage	Persistent edema, erythema
September 3rd Week (During HBOT)	Depth reduced, early granulation tissue	Erythema reduced; infection controlled	Granulation emerging, fibrin visible	Defined margins; wound contraction noted	Decreased edema, improved vascularity
October 2024 (Final HBOT Session)	Shallow wound; over 90 % epithelialized	No signs of active infection	Mature granulation; extensive epithelialization	Nearly closed wound; stable moisture	Minimal edema, healthy tissue borders

## Footnotes

1 Wound Zalf is a commercial antimicrobial ointment containing bacitracin and polymyxin B.
